# Robust Object Tracking Based on Motion Consistency

**DOI:** 10.3390/s18020572

**Published:** 2018-02-13

**Authors:** Lijun He, Xiaoya Qiao, Shuai Wen, Fan Li

**Affiliations:** Department of Information and Communication Engineering, School of Electronic and Information Engineering, Xi’an Jiaotong University, Xi’an 710049, China; jzb2016125@mail.xjtu.edu.cn (L.H.); qxy0212@stu.xjtu.edu.cn (X.Q.); wen201388@stu.xjtu.edu.cn (S.W.)

**Keywords:** object tracking, motion consistency, state prediction, position factor, occlusion factor

## Abstract

Object tracking is an important research direction in computer vision and is widely used in video surveillance, security monitoring, video analysis and other fields. Conventional tracking algorithms perform poorly in specific scenes, such as a target with fast motion and occlusion. The candidate samples may lose the true target due to its fast motion. Moreover, the appearance of the target may change with movement. In this paper, we propose an object tracking algorithm based on motion consistency. In the state transition model, candidate samples are obtained by the target state, which is predicted according to the temporal correlation. In the appearance model, we define the position factor to represent the different importance of candidate samples in different positions using the double Gaussian probability model. The candidate sample with highest likelihood is selected as the tracking result by combining the holistic and local responses with the position factor. Moreover, an adaptive template updating scheme is proposed to adapt to the target’s appearance changes, especially those caused by fast motion. The experimental results on a 2013 benchmark dataset demonstrate that the proposed algorithm performs better in scenes with fast motion and partial or full occlusion compared to the state-of-the-art algorithms.

## 1. Introduction

Object tracking is an important application for video sensor signal and information processing, which is widely applied in video surveillance, security monitoring, video analysis, and other areas. Although numerous methods have been proposed, it is still a challenging problem to implement object tracking in particular scenes, such as sports scenes for player tracking and in security scenes for criminal tracking. These scenes are characterized by fast motion of the target, occlusion and illumination variation. Improving the accuracy and robustness of tracking in these particular scenes is an open problem.

Object tracking is the determination of the target state in continuous video frames. The particle filter is widely used in object tracking, which uses the Monte Carlo method to simulate the probability distribution and is effective in estimating the non-Gaussian and nonlinear states [1]. In the particle filter, the state transition model and the appearance model are two important types of probabilistic models. The state transition model is used to predict the current target state based on the previous target states, which can be divided into Gaussian distribution model and constant velocity model. Gaussian distribution model assumes that the velocities between adjacent frames are uncorrelated. Thus, the candidate samples are predicted by simply adding a Gaussian disturbance to the previous target state [2,3,4,5,6,7,8]. This method has been widely used due to its simplicity and effectiveness. The method performs well when the target moves short distances in random directions. However, in the presence of fast motion, a large variance and more particles are needed to avoid the loss of the true target, which will result in higher computational burden. The constant velocity model assumes a strong correlation between the velocity of the current state and those of previous states, and candidate samples are predicted from the previous states and velocities with the addition of a stochastic disturbance [9,10,11,12,13,14,15]. This effect can be regarded as adding a disturbance to a new state that is far away from the previous state in terms of velocity. Thus, when the velocity in the current frame is notably smaller than that in the previous frame, an improper variance of the disturbance will also cause the loss of the target. The appearance model is used to represent the similarity between the real state of the target and the observation of the candidates. It can be divided into generative model [8,15,16,17,18,19,20], discriminative model [21,22,23,24,25,26,27] and collaboration model [2,3,4,6,28,29,30]. Tracking based on generative model focuses on foreground information and ignores background information. The candidates are represented sparsely by a foreground template set and the candidate with the minimum reconstruction error can be selected as the target. Tracking based on discriminative model considers the target tracking process as a classification process, and a classifier is trained and updated online to distinguish the foreground target from the background. Tracking based on collaboration model takes advantage of the generative and discriminative models to make joint decisions. Most existing target tracking methods have obvious disadvantages in complex scenes. The candidate samples selected in the state transition model without prediction may not include the real target, which might cause tracking drift or even tracking failure especially when the target has fast motion in the video. Moreover, if the target’s appearance changes drastically during movement, only considering the target’s appearance feature in appearance model may reduce the matching accuracy between the target and the candidate samples.

In this paper, we propose an object-tracking algorithm based on motion consistency (MCT). MCT utilizes the temporal correlation between continuous video frames to describe the motion consistency of the target. In the state transition model, the candidate samples are selected based on the predicted target state. In the appearance model, the candidate samples are represented by the position factor, which is defined to describe the motion consistency of the target. The tracking result is determined by combining the position factor with the local and holistic responses.

The main contributions of our proposed algorithm are summarized as follows:

1. Candidate sampling based on target state prediction

A conventional transition model may result in loss of the target if the number of candidate samples is limited. This may lead to tracking drift or even tracking failure. In this paper, candidate sampling based on target state prediction is proposed. Considering the motion consistency, the target state in the current frame is predicted based on the motion characteristics of the target in previous frames. Then, the candidate samples are selected according to the predicted target state to overcome the tracking drift.

2. Joint decision by combining the position factor with local and holistic responses

The appearance probability of the target may differ in different positions due to the motion consistency. Different from conventional algorithms, which neglect the temporal correlation of the video frames in the appearance model, the position factor is defined to characterize the importance of candidate samples in different positions according to the double Gaussian probability model. Meanwhile, the occlusion factor is proposed to rectify the similarity computed in the local representation to obtain the local response, and the candidates are represented sparsely by positive and negative templates in the holistic representation to obtain the holistic response. Then, the tracking decision is made by combining the position factor with the responses of the local and holistic representations. This approach makes full use of the temporal correlation of the target state and the spatial similarity of local and holistic characteristics.

3. Adaptive template updating based on target velocity prediction

The fast motion of the target will inevitably bring about appearance changes of the target and background. To adapt to the changes, an adaptive template updating approach is proposed, which is based on the predicted velocity of the target. The positive template set is divided into dynamic and static template sets. When the target moves fast, the dynamic positive template set is updated to account for the appearance changes of the target. To keep the primitive characteristics of the target, static positive template set is retained. Moreover, the negative holistic template set is updated continuously to adapt to the background changes.

The rest of the paper is organized as follows: Section 2 reviews the related work. In Section 3, an object tracking algorithm based on motion consistency is proposed. Section 4 gives the experimental results with quantitative and qualitative evaluations. This paper’s conclusions are presented in Section 5.

## 2. Related Work

A large number of algorithms have been proposed for object tracking, and an extensive review is beyond the scope of this paper. Most existing algorithms mainly focus on both state transition model and appearance model based on particle filter. In state transition model, the change in the target state is estimated and candidate samples are predicted with various assumptions. Most of the state transition models based on particle filter can be divided into Gaussian distribution model and constant velocity model, according to the assumption on whether the velocity is temporally correlated with previous frames. Appearance models, which are used to represent the similarity between the real state of the target and the observation of the candidates, can be categorized into generative models, discriminative models and combinations of these two types of models.

### 2.1. State Transition Models

The state transition model simulates the target state changes with time by particles prediction. Most of the algorithms use a Gaussian distribution model to predict candidate samples. The Gaussian distribution model regards previous target state as the current state, and then obtains candidate samples by adding a Gaussian noise dispersion to the state space. For example, in [2,3], the particles are predicted by a Gaussian function with fixed mean and variance, thus the coverage of particles is limited. It describes target motion best for short-distance motion with random directions, but it performs poorly when the target has a long-distance motion in a certain direction. Furthermore, in [5], three types of particles whose distributions have different variances are prepared to adapt to the different tasks. In visual tracking decomposition [7], the motion model is represented by a combination of multiple basic motion models, each of which covers a different type of motion using a Gaussian disturbance with a different variance. To a certain extent, the model can cover abrupt motion with a large variance. However, fixed models cannot adapt to complex practical situations. In addition, there are other algorithms which ignore the temporally correlation of the target in continuous frames. For example, multi-instance learning tracker (MIL) [22] utilizes a simplified particle filtering method which sets a searching radius around the target in the previous frame and uses dense sampling to obtain samples. Small searching radius cannot adapt to fast motion, on the other hand large searching radius will bring high computation burden. Since algorithms [20,31] based on local optimum search obtain the candidates without prediction either, thus a larger searching radius is also necessary when fast motion occurs.

In subsequent studies, some of the algorithms use the constant velocity model to predict candidate samples, which assumes that the velocity of the target in the current frame is correlated to that in the previous frame. In [9,10], the motion of the target is modeled by adding the motion velocity with the frame rate (motion distance) in the previous frame to the previous state and then disturbing it with Gaussian noise. In [11,12], the average velocity of the target in previous frames is considered to obtain the translation parameters to model the object motion. This is suitable for the situation that the velocity in the current frame is strongly correlated with that in the previous frame. However, the actual motion of the target is complex. When the target in the current frame is not correlated with that in the previous frame, the improper variance of Gaussian disturbance and finite number of particles will also lead to tracking drift or even failure.

### 2.2. Appearance Models

Tracking based on generative models focuses more on foreground information. The candidates are represented by the appearance model and the candidate with the minimum reconstruction error can be selected as the target. In [8], multi-task tracking (MTT) represents each candidate by the same dictionary and considers each representation as a single task, and then formulates object tracking as a multi-task sparse learning problem by joint sparsity. In [19], a local sparse appearance model with histograms and mean shift is used to track the target. Tracking based on the L1 minimum (L1APG) in [12] models target appearance by target templates and trivial templates with sparse representation. However, it neglects the background information. Thus, in the presence of background cluster or occlusion, tracking based on generative model cannot distinguish the target from the background.

Tracking based on discriminative models, in essence, considers the target tracking process as a classification task and distinguishes the foreground target from the background using the classifier with the maximum classifier response. In [24,25], an online boosting classifier is proposed to select the most discriminating features from the feature pool. In [26], the supervised and semi-supervised classifiers are trained on-line, in which the labeled first frame sample and unlabeled subsequent training samples are used. The discriminative model considers the background and foreground information completely to distinguish the target. However, a classifier with an improper updating scheme or limited training samples will lead to bad classification.

Tracking methods that combine generative and discriminative models exploit the advantages of these two models, fusing them and using them together to make decisions. Tracking via the sparse collaborative appearance model (SCM) [2] combines a sparse discriminative classifier with a sparse generative model to track the object. With the sparse discriminative classifier, the candidate samples are represented sparsely by holistic templates and the confidence value is computed. In sparse generative model, the candidate samples are divided into patches and represented by a dictionary to compute the similarity between each candidate sample and the template. However, the positive templates remain; thus the discriminative classifier cannot adapt to drastic appearance variations. In object tracking via key patch sparse representation [4], the overlapped patch sampling strategy is adopted to capture the local structure. In the occlusion prediction scheme, the positive and negative bags are obtained by sampling patches within and outside the bounding box. Then the SVM (support vector machine) classifier is trained by the bags with labels, by which the patches are predicted to be occluded or not. Meanwhile, the contribution factor is computed via sparse representation. However, the SVM classifier is not timely updated, which may cause error accumulation in occlusion decisions. In object tracking via a cooperative appearance model [6], an object tracking algorithm based on cooperative appearance model is proposed. The discriminative model is established in local representation with positive and negative dictionaries. The reconstruction error of each candidate is computed in holistic representation, and the impact regions are proposed to combine the local and holistic responses.

In this paper, we propose a tracking algorithm based on motion consistency, in which the candidate samples are predicted by a state transition model based on target state prediction. In addition, in the appearance model, the position factor is proposed to assist in target selection. As revealed in [2,3,5,7], the candidate samples are predicted simply by using Gaussian distribution model, which is effective for short-distance motion with random direction. Then, the algorithms in [9,10,11,12] take the velocity of the target into consideration, to a certain extent, which can adapt to motion that is correlated to the previous state, even long-distance motion. Inspired by the above work, in this paper, the predicted target state information is added to the conventional Gaussian distribution model. However, the proposed model is different from the constant velocity model in that only dynamic candidate samples are predicted with motion prediction; also, some basic candidate samples around the previous state are predicted with Gaussian disturbance. In this way, varied candidate samples can adapt to complex motions in addition to fast motion. Conversely, most tracking algorithms focus on the similarity between the candidate samples and templates, such as that in [2]. However, when the target appearance has a large variation, the true target cannot be selected by only considering the similarity. Thus, in the appearance model, considering the temporal correlation of the target state, the position factor is proposed to characterize the importance of candidate samples in different positions and collaborates with representation responses to select the target.

## 3. Robust Object Tracking Based on Motion Consistency

Conventional algorithms which ignore the temporal correlation of the target states in state transition model may lose the true target, especially when fast motion occurs, and if the appearance of the target changes drastically, algorithms that only focus on the target’s appearance feature in the appearance model will struggle to separate foreground targets from the background. Meanwhile, an appropriate updating scheme is necessary to maintain the robustness of the algorithm. Thus we propose a robust object tracking algorithm based on motion consistency.

**Figure 1:**
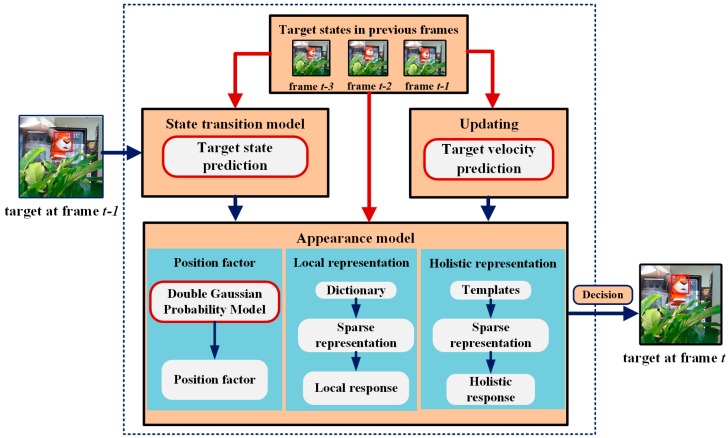
The candidates are predicted by the target state prediction based state transition model. In appearance model, the position factor is proposed to characterize the importance of candidate samples in different positions. In the target velocity prediction based updating model, the template sets are updated adaptively to ensure the robustness and effectiveness of MCT algorithm.

The tracking process of our proposed MCT algorithm is shown in Figure 1. The target is assumed to be known in the first frame. In the state transition model, the target state, including the motion direction and distance in the current frame, is predicted according to the target states in previous frames. The candidate samples are predicted by the state transition model according to the predicted target state. Next, all the candidate samples are put into the appearance model. In the appearance model, we propose the position factor according to the double Gaussian probability model to assign weights to candidate samples located in different positions. Meanwhile, in the local representation, the similarity between the candidate and the target is computed with the sparse representation by dictionary to obtain the local response of each candidate. In the holistic representation, the holistic response of each candidate sample is computed with the sparse representation by positive and negative templates. Finally, the candidate sample with the highest likelihood is decided as the tracking result by combining the holistic and local responses with the position factor. To adapt to the appearance changes of the target, especially those caused by fast motion, the template sets should be updated adaptively based on the target velocity prediction.

### 3.1. State Transition Model Based on Target State Prediction

The state transition model is used to select the candidate samples in the current video frame. Conventional methods based on particle filter usually use the Gaussian distribution model to obtain the candidate samples around the position where the target appears in the previous frame. When the target has fast motion, the selection of candidate samples without prediction may result in the loss of the true target. Therefore, considering the characteristics of motion consistency, we propose a state transition model based on target state prediction to predict candidate samples.

#### 3.1.1. Target State Prediction

**Figure 2:**
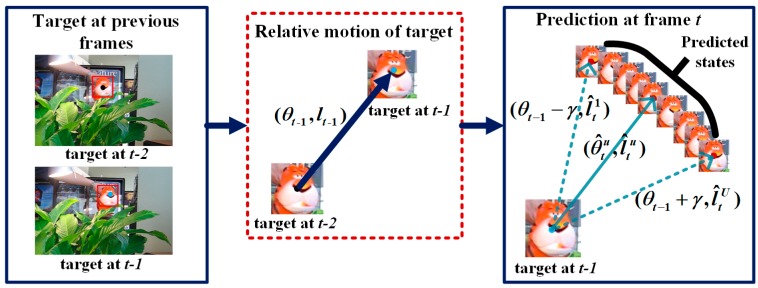
The relative motion of target in the previous frame including motion direction and motion distance is already known. Then the target motion direction in the current frame is predicted by adding a prediction range of *γ* to the previous motion direction, and the motion distance is predicted according to the previous motion distance with variation rate.

Target state prediction is the estimation of the target position at frame t. We predict a set of target motion directions and distances $\left\{\left({\hat{\theta }}_{t}^{1},{\hat{l}}_{t}^{1}),({\hat{\theta }}_{t}^{2},{\hat{l}}_{t}^{2}),\cdots ,({\hat{\theta }}_{t}^{u},{\hat{l}}_{t}^{u}),\cdots ,({\hat{\theta }}_{t}^{U},{\hat{l}}_{t}^{U}\right)\right\}$ at frame *t* according to the motion consistency, which is shown in Figure 2.

It is assumed that the target has a steady motion direction and velocity. We predict the target motion direction by using the motion direction of the target at frame *t* − 1. As shown in Figure 2, the relative motion direction from the target from frame *t* − 1 to frame *t* − 2 is *θ_t_*_−1_. Then, the target motion direction is predicted as a set of values based on the value *θ_t_*_−1_. Taking *θ_t_*_−1_ as the center, we select *U* angles uniformly in the interval [*θ_t_*_−1_ − *γ*, *θ_t_*_−1_ + *γ*], where *γ* is a constant, to determine the prediction range to reduce the estimation error. Then, the predicted values of the motion directions are:

(1) $${\hat{\theta }}_{t}^{u}=\theta_{t-1}-\gamma +\frac{2\gamma }{U-1}(u-1)\;u=1,2,\ldots ,U,$$

where *U* is the number of predicted directions.

We also predict the motion distance based on the relative motion distance within the three frames before frame. The predicted distance in each direction is:

(2) $${\hat{l}}_{t}^{u}={\hat{w}}_{t}\times l_{t-1},$$

where $l_{t-1}$ is the relative motion distance of the target from frame *t* − 1 to frame *t* − 2. ${\hat{w}}_{t}$ describes the variation rate of the motion distance in the previous frames and is defined as:

(3) $${\hat{w}}_{t}=\alpha \times \frac{l_{t-1}}{l_{t-2}}+(1-\alpha )\times \frac{l_{t-2}}{l_{t-3}},$$

where α is a positive constant that is less than 1.

Thus, we obtain a set of target motion directions and distances $\left\{\left({\hat{\theta }}_{t}^{1},{\hat{l}}_{t}^{1}),({\hat{\theta }}_{t}^{2},{\hat{l}}_{t}^{2}),\cdots ,({\hat{\theta }}_{t}^{u},{\hat{l}}_{t}^{u}),\cdots ,({\hat{\theta }}_{t}^{U},{\hat{l}}_{t}^{U}\right)\right\}$.

#### 3.1.2. Three-Step Candidate Sampling

**Figure 3:**
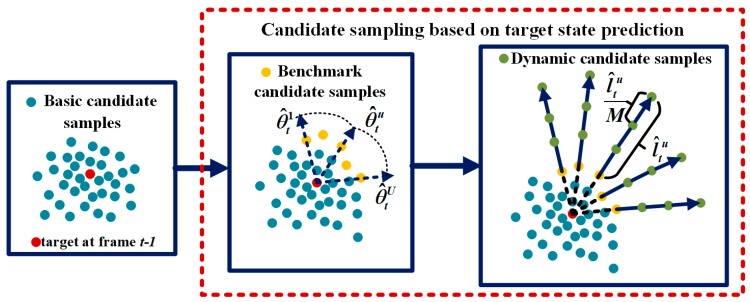
First, the basic candidate samples (the blue dots) are obtained around the target location at frame *t* − 1. Second, the basic candidate samples with the largest relative distance in each predicted direction will be selected as the benchmark candidate samples (the yellow dots). Third, adding more dynamic candidate samples (the green dots) in the predicted direction begins from the benchmark within a predicted distance.

The conventional Gaussian distribution model generates the candidate samples near the target position in the previous frame. For random motion over a short distance, the true target position can be successfully covered by the samples. However, when fast motion occurs, these predicted samples may lose the true target position, which results in tracking drift or tracking failure. To avoid the loss of the true target position, a three-step candidate sampling scheme is proposed according to target state prediction, which is illustrated in Figure 3.

1. First step: Basic candidate sampling

The basic candidate samples in the current frame are predicted by the Gaussian distribution model. The current state $X_{t}^{i}$ is predicted by the state in the previous frame $X_{t-1}^{i}$ and the disturbance by Gaussian noise $X_{Gauss}$ as follows:

(4) $$X_{{}_{t}}^{i}=X_{{}_{t-1}}^{i}+X_{Gauss},$$

where $X_{t}^{i}$ represents the *i*th candidate in frame t and the state of each candidate is represented by an affine transformation model of six parameters $X_{t}^{i}=\left(\alpha_{1}^{i},\alpha_{2}^{i},\alpha_{3}^{i},\alpha_{4}^{i},\alpha_{5}^{i},\alpha_{6}^{i}\right)$, in which $\left(\alpha_{1}^{i},\alpha_{2}^{i}\right)$ represents translation, $\left(\alpha_{3}^{i},\alpha_{4}^{i}\right)$ represents scaling and $\left(\alpha_{5}^{i},\alpha_{6}^{i}\right)$ represents rotation. Each parameter in the state vector $\left(\alpha_{1}^{i},\alpha_{2}^{i},\alpha_{3}^{i},\alpha_{4}^{i},\alpha_{5}^{i},\alpha_{6}^{i}\right)$ is assumed to obey a Gaussian distribution of different mean and variance according to the actual situation. Next, the relative translation between the *N* basic candidate samples and the target in the previous frame is computed and converted to a polar coordinate form $\left\{\left(\theta_{t}^{1},l_{t}^{1}),(\theta_{t}^{2},l_{t}^{2}),\cdots ,(\theta_{t}^{n},l_{t}^{n}),\cdots ,(\theta_{t}^{N},l_{t}^{N}\right)\right\}$, which covers the direction and distance information of each candidate sample.

2. Second step: Benchmark candidate sampling

To make candidate samples cover as large a region as possible where the target possibly appears, considering both efficiency and effectiveness, we simplify the problem by selecting the benchmark with the largest relative distance in each predicted direction and then adding more dynamic candidate samples in the same direction within a predicted distance, which begins from the benchmark.

The benchmark candidate samples $\left\{\left({\hat{\theta }}_{t}^{1},l_{\max}^{1}),({\hat{\theta }}_{t}^{2},l_{\max}^{2}),\cdots ,({\hat{\theta }}_{t}^{u},l_{\max}^{u}),\cdots ,({\hat{\theta }}_{t}^{U},l_{\max}^{U}\right)\right\}$ are selected from the basic candidate samples in accordance with the motion direction predicted by Equation (1) as follows:

(5) $$({\hat{\theta }}_{t}^{u},l_{\max}^{u})=(\theta_{t}^{j},l_{t}^{j})\;\;\;\;j=\underset{i\in \{i\;|\;\theta_{t}^{i}={\hat{\theta }}_{t}^{u},1\leq i\leq N\}}{\arg\max}l_{t}^{i}\;\;\;\;u=1,2,\cdots ,U,$$

In the ${\hat{\theta }}_{t}^{u}$ direction, the basic candidate sample with the largest relative distance will be selected as the benchmark candidate, as shown in Figure 3. There is a rule that if it lacks a benchmark in the ${\hat{\theta }}_{t}^{u}$ direction, the benchmark candidate sample will be defined as $\left({\hat{\theta }}_{t}^{u},0\right)$ with a relative distance of zero.

3. Third step: Dynamic candidate sampling

Beginning from the benchmark, in each predicted direction, a total of *M* dynamic candidate samples is placed evenly at a distance of ${\hat{l}}_{t}^{u}/M$ apart, as shown in Figure 3. There are *UM* dynamic candidate samples:

(6) $$({\hat{\theta }}_{t}^{u},l_{\max}^{u}+m\times \frac{{\hat{l}}_{t}^{u}}{M})\;\;\;\;u=1,2,\cdots ,U\;\;\;\;m=1,2,\cdots ,M,$$

Thus, we obtain the relative position information of all the candidate samples including *N* basic candidate samples and *UM* dynamic candidate samples $\left\{\left(\theta_{t}^{1},l_{t}^{1}),(\theta_{t}^{2},l_{t}^{2}),\cdots ,(\theta_{t}^{N},l_{t}^{N}),(\theta_{t}^{N+1},l_{t}^{N+1}),\cdots ,(\theta_{t}^{N+UM},l_{t}^{N+UM}\right)\right\}$ in the frame t. Moreover, these relative polar coordinates are converted into translation parameters, and the remaining four parameters are assumed to obey the Gaussian distribution. All the candidate samples predicted in frame *t* are represented by $X_{t}=\left\{X_{t}^{1},X_{t}^{2},\cdots ,X_{t}^{N+UM}\right\}$.

### 3.2. Position Factor Based Cooperative Appearance Model

In the presence of fast motion, the target’s appearance changes drastically. Therefore, the algorithms that only considering the changes of appearance feature in appearance model is difficult to distinguish the foreground target from a complex background. In view of this, we propose the position factor to reduce the effect of appearance changes caused by target movement. Due to the motion consistency, there is a different probability of the target appearing in each position. Generally, candidate sample in the location where the target may appear has a larger probability to be selected as the target. Thus, candidate samples of this kind should be assigned larger weights, while other candidate samples should be assigned smaller weights. Therefore, a quantitative parameter named the position factor is proposed to assign different weights to candidate samples in different positions to rectify the representation error. The likelihood of candidate sample is defined by combining the position factor with the responses of local and holistic representations.

#### 3.2.1. Position Factor Based on Double Gaussian Probability Model

The position factor of the *i*th candidate sample is computed by multiplying the direction score $S_{A}^{i}$ and distance score $S_{l}^{i}$:

(7) $$F^{i}=S_{A}^{i}S_{l}^{i}\;\;\;\;i=1,2,\cdots ,N+MU,$$

To compute direction and distance scores, a double Gauss probability model is used according to the target states in previous frames, which includes the direction Gaussian probability model and distance Gaussian probability model.

1. Direction score

The motion direction of the target at frame *t* is consistent with that in the previous frame. It is assumed that the probability of the target appearing in the $\theta_{t-1}$ direction is the largest, and the probability of the target appearing in the opposite direction $\theta_{t-1}$ is the smallest. Thus, a Gaussian probability model with $\theta_{t-1}$ mean error is proposed to simulate the distribution of the target’s motion direction. Accordingly, the probability of the candidate sample lying in $\theta_{t-1}$ being selected as the target is large and it should be assigned a large weight. In contrast, the candidate sample lying in the direction opposite $\theta_{t-1}$ should be assigned a small weight. Then, the weight assignment model is transformed according to the trend of the direction Gaussian probability model, from which the direction score of the *i*th candidate sample is derived as follows:

(8) $$S_{A}^{i}=\frac{T_{1}}{1-e^{-\pi }}e^{-{\left(\theta_{t}^{i}-\theta_{t-1}\right)}^{2}}+(1-\frac{T_{1}}{1-e^{-\pi }})\;\;\;\;i=1,2,\cdots ,N+MU,$$

where $\theta_{t}^{i}$ denotes the relative direction between the *i*th candidate and target at frame t−1, $\theta_{t-1}$ is the motion direction of the target at frame t−1, and $T_{1}$ is a constant that depends inversely on $\left(\theta_{t-1},p_{\max}^{A}\right)$ and $\left(\theta_{t-1}-\pi ,p_{\min}^{A}\right)$.

2. Distance score

The motion velocities within two continuous frames are consistent. In the $\theta_{t-1}$ direction, the velocity of the target at frame t is consistent with that of the previous frame. Thus, according to the distance predicted by Equation (2), it is assumed that the probability of the target appearing near the relative distance ${\hat{l}}_{t}^{u}$ is the largest. Conversely, in the direction opposite $\theta_{t-1}$, the motion velocity of the target is small, and the probability of the target at frame t appearing near the target at frame t−1 is the largest. Accordingly, in each direction, there is a distance with the largest probability of target appearance, as well as directions where the candidate samples are located:

(9) $$l_{}^{i}=(\frac{T_{2}}{1-e^{-\pi }}e^{-(\theta_{t}^{i}-\theta_{t-1}{)}^{2}}+(1-\frac{T_{2}}{1-e^{-\pi }})){\hat{l}}_{{}_{t}}^{u}\;\;\;\;i=1,2,\cdots ,N+MU,$$

where $T_{2}$ is a constant that depends inversely on $\left(\theta_{t-1},{\hat{l}}_{t}^{u}\right)$ and $\left(\theta_{t-1}-\pi ,k{\hat{l}}_{t}^{u}\right)$, and *k* is a positive constant that is less than 1.

It has been assumed that in the $\theta_{t}^{i}$ direction, the probability of the target appearing at the relative distance $l_{}^{i}$ is largest and smallest for the infinite relative distances. Thus, the probability of the candidate sample at $\left(\theta_{t}^{i},l_{}^{i}\right)$ being selected as the target is large and the weight of this candidate is defined as $p_{\max}^{l}$, whereas the probability of the candidate sample at $\left(\theta_{t}^{i},\infty \right)$ being selected as the target is small and the weight of this candidate is defined as $p_{\min}^{l}$. According to this distribution feature, in the $\theta_{t}^{i}$ direction, the motion distance can be assumed to obey a Gaussian probability model with $l_{}^{i}$ mean error. Thus, the weight assignment model is transformed according to the trend of the distance Gaussian probability model, by which the distance score of the *i*th candidate sample is computed as follows:

(10) $$S_{l}^{i}=\frac{T_{3}}{1-e^{-{\hat{l}}_{i}^{u}}}e^{-{\left(l_{t}^{i}-l_{}^{i}\right)}^{2}}+(1-\frac{T_{3}}{1-e^{-{\hat{l}}_{i}^{u}}})\;\;\;\;i=1,2,\cdots ,N+MU,$$

where $l_{t}^{i}$ represents the relative distance of the *i*th candidate sample; $l_{}^{i}$ is the corresponding distance with the largest probability in the $\theta_{t}^{i}$ direction, which is computed by Equation (9); and $T_{3}$ is a constant that depends inversely on $\left(l_{}^{i},p_{\max}^{l}\right)$ and $\left(\infty ,p_{\min}^{l}\right)$.

#### 3.2.2. Local Representation with Occlusion Factor

In the local representation, the candidate samples are divided into patches and are sparsely represented by the local features, from which the local information of candidate samples is obtained. The local information of candidates, obtained from the sparse representation, is used for adapting to occlusion and other situations that may cause local appearance changes. Thus, in this paper, based on the sparse generative model (SGM) [2], the occlusion information of each patch is defined according to the reconstruction error with the local sparse representation. Furthermore, to adapt to occlusions, the occlusion factor is defined to rectify the similarity computed by SGM. Thus, the local response *L* of each candidate is represented as follows:

(11) $$L=L_{c}^{}\beta_{c}^{},$$

where $L_{c}^{}$ represents the similarity and $\beta_{c}^{}$ represents the occlusion factor.

1. Similarity function

First, the image is normalized to 32 × 32 pixels, from which *M* patches are derived by overlapping sliding windows. With the sparse representation, the patches are represented by a dictionary generated from the first frame by K-Means [32] to compute the reconstruction error. The patch with large reconstruction error is regarded as an occluding part and the corresponding element of the occlusion indicator o is set to zero, as follows:

(12) $$o_{m}=\left\{ \begin{array}{l} 1\;\;\;\;\varepsilon_{m}<\varepsilon_{0}\\ 0\;\;\;\;otherwise \end{array}\right.\;\;\;\;m=1,2,\cdots ,M,$$

where $\varepsilon_{m}$ denotes the reconstruction error of *m*th patch and $\varepsilon_{0}$ is a constant used to determine whether the patch is occluded or not.

Moreover, a weighted histogram is generated by concatenating the sparse coefficient with the indicator *o*. The similarity $L_{c}$ of each candidate is computed on the basis of weighted histograms between the candidate and the template.

2. Occlusion factor

To further adapt to occlusion in the appearance model, we propose the occlusion factor to rectify the similarity function. The number of the patches whose reconstruction errors are larger than $\varepsilon_{0}$ is computed statistically by:

(13) $$N_{c}^{}=\sum_{m=1}^{M}o_{m},$$

where a larger value of $N_{c}$ represents that there is more information on the background or the occlusion. The occlusion factor is defined as follows:

(14) $$\beta_{c}=\sigma e^{-(1-\omega_{c})},$$

where σ is a constant and $\omega_{c}=N_{c}/M$ is the statistical average of $N_{c}$. According to Equation (14), there is a positive correlation between the occlusion factor and $\omega_{c}$. When occlusion occurs, the value of the occlusion factor increases with the increase of $\omega_{c}$.

#### 3.2.3. Holistic Representation Based on Sparse Discriminative Classifier

In the holistic representation, the foreground target is distinguished from the background by sparse discriminative classifier (SDC) [2]. Initially, *n_p_* positive templates are drawn around the target location and *n_n_* negative templates are drawn further away from the target location. By the way, the label +1 is assigned to positive templates and label −1 is assigned to negative templates. In order to reduce the redundancy of the grayscale template sets, a feature selection scheme is proposed in SDC. First, the labels are represented sparsely by the positive and negative template sets and a sparse vector **s** is generated by the sparse coefficient vector. Then the sparse vector **s** is constructed into a diagonal matrix S’ and a projection matrix **S** is obtained by removing all-zero rows from S’. Finally, both the templates and candidates are reconstructed into the projected form by combining them with the projection matrix **S**. With the sparse representation, the projected candidates are represented by projected template sets, and the reconstruction error of the projected candidate is used to compute the confidence value *Hc* of each candidate.

#### 3.2.4. Joint Decision by Combining Position Factors with Local and Holistic Responses

By combining the position factor with the local and holistic responses, the likelihood function of the *i*th candidate sample is computed as follows:

(15) $$p^{i}=F^{i}L^{i}H_{c}^{i}\;\;\;\;i=1,2,\cdots ,N+MU,$$

For each candidate, the position factor is large if it is located in the position where the target appears with the largest probability. Moreover, the local and holistic responses are large if the candidate is similar to the target. Thus, the candidate with the largest likelihood, calculated by Equation (15), will be possibly identified as the target. The tracking result *X_t_* in the current frame is chosen from the candidate samples by:

(16) $$X_{t}=X_{t}^{j}\underset{}{\;\;\;\;\;j=\underset{i=1,2,\cdots ,N+MU}{\arg_{i}\max}}p^{i},$$

### 3.3. Adaptive Template Updating Scheme Based on Target Velocity Prediction

It is necessary to update the templates and the dictionary to capture the appearance changes of the target to ensure the robustness and effectiveness of the appearance model. In SCM [2], the negative templates are updated in the SDC model every several frames from the image regions away from the current tracking result, but the positive templates remain. In the SGM model, instead of updating the dictionary, the template histogram is updated by taking the most recent tracking result into account.

However, the fixed positive templates in SDC model cannot match the true target effectively when the target’s appearance changes drastically, which will inevitably occur in the presence of fast motion. To adapt to the changes of the target’s appearance, the positive templates also need to be updated to capture the appearance changes of the target. In view of this, an adaptive template updating scheme based on the prediction of the target velocity is proposed.

The positive template set is divided into dynamic and static template sets, as follows:

(17) $$n_{p}=\{p_{d},\;p_{s}\}$$

where $p_{d}$ represents the dynamic template set and $p_{s}$ represents the static template sets. For every several frames, we determine whether the target has fast motion by comparing the relative motion distance of the target with a threshold of *p* pixels. The variable *p* denotes the fast motion measure of the tracking process. When the relative motion distance $l_{t}$ is larger than *p*, we deem that the target moves fast in the current frame, hence the target in the next frame will move with a large velocity according to motion consistency. Thus, the target may have appearance change at the same time. Next, the dynamic positive template set is updated to account for appearance changes of the target. To keep the initial information of the target and avoid incorrect representation in case of the recovery of target’s appearance, the static positive template set is retained, as follows:

(18) $$n_{p}=\left\{ \begin{array}{l} \{p_{d}^{*},\;p_{s}\}\;\;\;\;\;\;\;\;\;l_{t}\geq p\\ \{p_{d},\;p_{s}\}\;\;\;\;\;\;\;\;\;otherwise \end{array}\right.$$

where $p_{d}^{*}$ represents the updated dynamic template set, which is drawn around the target location in the current frame. By combining the updated dynamic set and static template set, the target can be well matched with the positive template set. Besides, the negative templates are also updated in the SDC model every several frames in order to distinguish the target from the complex and changeable background in the tracking process.

## 4. Experiments

To evaluate the performance of our proposed MCT algorithm, we test our tracker on the CVPR2013 (OTB2013) dataset, which has been widely used in evaluation [33]. OTB contains 50 video sequences, source codes for 29 trackers and their experimental results on the 50 video sequences. We compared our tracker with eight state-of-the-art tracking algorithms: the compressive tracking (CT) [23], distribution fields for tracking (DFT) [31], online robust image alignment (ORIA) [20], MTT [8], visual tracking via adaptive structural local sparse appearance model (ASLA) [18], L1APG [12], SCM [2], and MIL [22] trackers. The basic information of these trackers is listed in Table 1. As described in related work, most algorithms ignore the temporal correlation of the target states. MTT, ASLA and SCM obtain candidates by Gaussian distribution model, MIL and CT obtain candidates by dense sampling, DFT and ORIA obtain candidates by local optimum search. Only LIAPG considers target motion speed in the previous frame and obtain candidates by constant velocity model. Besides, the competition algorithms cover all the type of appearance model, but all of the appearance models are designed from the perspective of the target’s appearance feature. We evaluate the CT, DFT, ORIA, MTT, ASLA, L1APG, SCM, and MIL trackers by using the available source codes. We conduct experiments on all 50 video sequences and perform a concrete analysis on eight specific video sequences with different problems. All the problems we concerned are covered in the video sequences, thus the effectiveness of the algorithm can be illustrated. The basic information of the eight test video sequences and primary problems are shown in Table 2 [33].

In the experiment, for each test video sequence, all information is unknown except for the initial target position of the first frame. The number of candidate samples is 600 including 250 basic candidate samples and 350 dynamic candidate samples. The number of motion directions we predicted is seven. In each direction, a total of 50 candidate samples are obtained. In the update, five of positive templates are updated every five frames from image regions away more than 20 pixels the current tracking result.

### 4.1. Quantitative Evaluation

#### 4.1.1. Evaluation Metric

We choose three popular evaluation metrics: center location error, tracking precision and success rate.

The center location error (CLE) is defined as the Euclidean distance between the center position of the tracking result and the ground truth. Lower average center location error indicates better performance of the tracker. CLE is calculated by:

(19) $$\mathrm{CLE}=\frac{1}{N}\sum_{i=1}^{N}dis\left(C_{t}^{i},C_{gt}^{i}\right),$$

where N is the total number of frames, dis(,) represents distance between the center position $C_{t}^{i}$ of the tracking result and the ground truth $C_{gt}^{i}$ in the *i*th frame.

The tracking precision is defined as the ratio of the number of frames for which the error between the target center point calculated by the tracking algorithm and the groundtruth is smaller than the threshold *th* to the total number of frames, as follows:

(20) $$precision(th)=\frac{1}{N}\sum_{i=1}^{N}F(dis(C_{t}^{i},C_{gt}^{i})<th),$$

where F(·) is a Boolean function, the output of which is 1 when the expression inside brackets is true, otherwise the output is 0, and the threshold th∈[0,100]. The tracking precision depends on the choice of *th*. The threshold *th* is set to 20 to compute the tracking precision in the following experiment.

The success rate is defined as the ratio of the number of frames for which the overlap rate is larger than λ to the total number of frames, as follows:

(21) $$success(\lambda )=\frac{1}{N}\sum_{i=1}^{N}F(overlap^{i}\geq \lambda ),$$

where the overlap rate is defined as $overlap=\left|\tau_{gt}\cap \tau_{t}\right|/\left|\tau_{gt}\cup \tau_{t}\right|$, the threshold λ∈[0,1] is fixed, $\tau_{gt}$ represents the region inside the true tracking box generated by ground truth, $\tau_{t}$ represents the region inside the bounding box tracked by the tracker, |·| denotes the pixels in the region, and ∩ and ∪ denote the intersection and union of two regions, respectively. In the following experiment, instead of using the success rate value for a specific threshold λ, the area under the curve (AUC) is utilized to evaluate the algorithms.

#### 4.1.2. Experiment on CVPR2013 Benchmark

To evaluate the performance of our algorithm, we conduct an experiment to test the MCT tracker on the OTB dataset. OTB contains 50 videos and 29 algorithms, along with their experimental results on the 50 video sequences. We compared our tracker with eight trackers in terms of overall performances and we especially focus on the performance in the presence of fast motion (FM) and occlusion (OCC).

1. The comparison results for fast motion and occlusion

**Figure 4:**
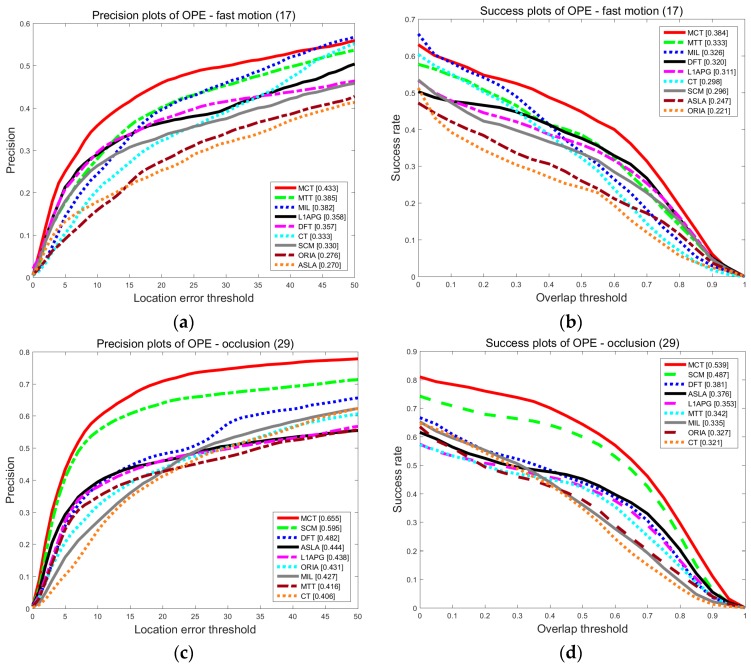
The precision plot (**a**) and the success rate plot (**b**) of the tracking results on OTB for FM, and the precision plot (**c**) and the success rate plot (**d**) of the tracking results on OTB for OCC.

Fast motion (FM) and occlusion (OCC) are the two scenarios that we examine. The results of precision and success rate for FM and OCC are shown as Figure 4. Our algorithm has the highest precision and success rate compared with others. In the presence of FM, the tracking precision of MCT is 0.433, which is 10% higher than that of the SCM algorithm. The success rate is 0.384, which is improved by 8.8% compared with the SCM algorithm. According to the motion consistency, the target state is estimated and merged into the state transition model, appearance model and updating scheme to promote the accuracy in the presence of fast motion. In the presence of OCC, the tracking precision of MCT is 0.655, which is 6% higher than that of the SCM algorithm. The success rate is 0.539, which is 5.2% higher than that of the SCM algorithm. The performance of MCT is still the best in the presence of OCC. This finding is attributable to the occlusion factor, which is proposed to rectify holistic and local responses to account for OCC.

2. The comparison results for overall performance

**Figure 5:**
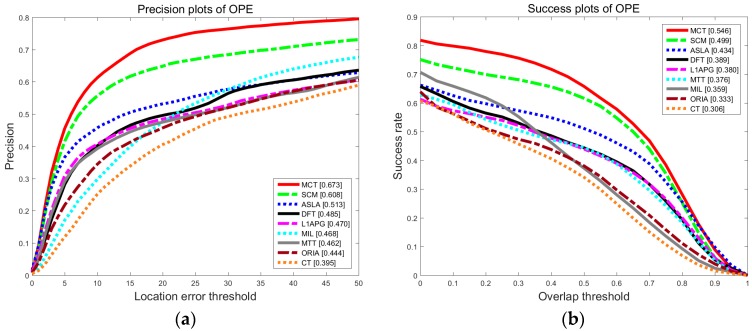
The precision plot (**a**) and the success rate plot (**b**) of the tracking results on OTB for overall performances.

The results of precision and success rate for overall performance on 50 video sequences are shown as Figure 5. Compared with other algorithms, the precision and the success rate of MCT are highest. The precision of MCT is 0.673, which is 6.5% higher than that of SCM. The success rate is 0.546, which is improved by 4.7% compared with the SCM algorithm. Compared with the other eight trackers, MCT has the best performance.

#### 4.1.3. Quantitative Evaluation on Eight Specific Video Sequences

The following experiment is conducted on eight specific video sequences, namely, *Basketball*, *Boy*, *Football*, *Jogging-1*, *Girl*, *Skating1*, *Coke*, and *Sylvester*, and the performances of the algorithms are evaluated on the metrics of center location error, tracking precision and success rate.

1. Center location error comparison

**Figure 6:**
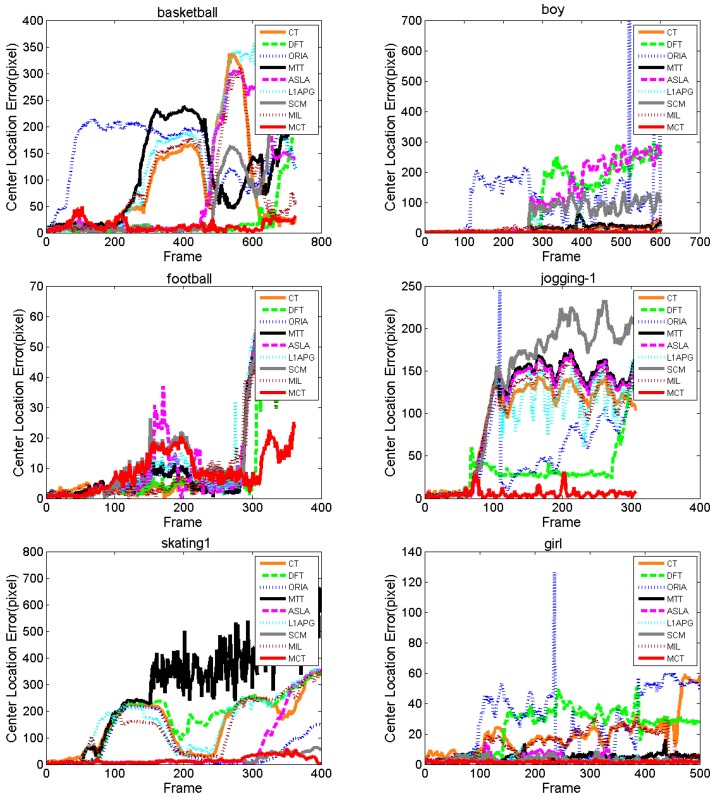

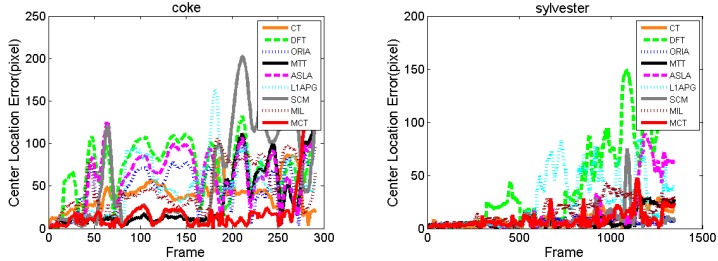
Center location error comparison of the algorithms.

The comparison of the center location errors is shown as Figure 6. For *Basketball*, the CLE of ORIA increases at approximately the 50th frame, and those of CT, MTT, L1APG and MIL increase at approximately the 200th frame. The CLEs of ASLA and SCM increase at approximately the 500th frame. DFT performs similarly to MCT before the 650th frame, but after the 650th frame, DFT has a sharply increasing CLE. Almost all the methods fluctuate violently with a high CLE because the basketball player moves in a random manner with deformation and illumination variation. The CLE of MCT between the 80th frame and the 200th frame fluctuates, but it recovers soon after with a low CLE. For *Boy*, the CLE of ORIA increases at approximately the 100th frame because the motion blur begins. The CLEs of DFT, ASLA and SCM increase at approximately the 260th frame and cannot resume tracking again because the boy moves fast with deformation, motion blur and scale variation. MCT performs better with smaller fluctuations compared with CT, MTT, L1APG and MIL. For *Football*, almost all the algorithms have a dramatic fluctuation at approximately the 170th frame because of deformation, but the CLE of MCT is the lowest. For *Jogging-1*, only MCT is stable with the lowest CLE. For *Coke*, partial and full occlusions appear from approximately the 30th frame, and the accuracies of the algorithms decrease, except for MCT. For *Skating1*, *Girl* and *Sylvester*, MCT maintains a stable performance with the lowest CLE.

Table 3 lists the average center location errors of the eight algorithms. Red indicates that the algorithm has the lowest CLE with the best performance followed by blue and green. We can see that the MCT algorithm has the lowest CLE on seven of these tests and the lowest average CLE on all tests. To sum up, by the CLE comparison with other algorithms, we can determine that our proposed algorithm, MCT, outperforms the others under different challenges.

2. Tracking precision comparison

**Figure 7:**
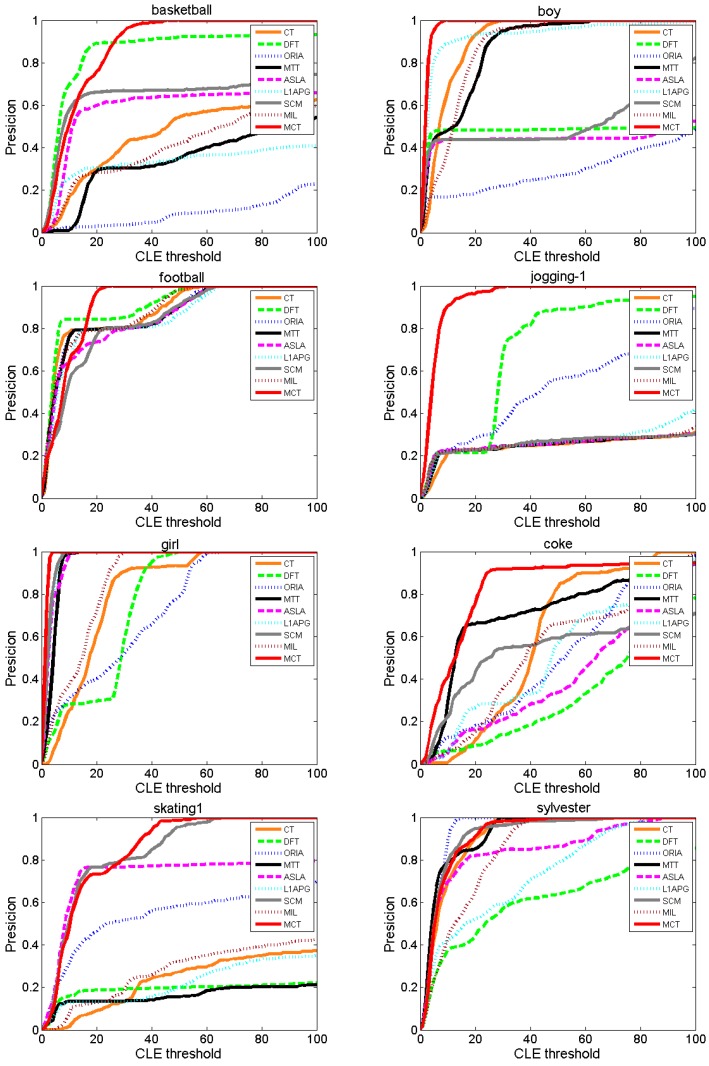
Precision comparison of the algorithms.

Figure 7 shows the precision results of each algorithm for various values of the threshold *th* within [0, 100]. MCT is the first algorithm to reach 100% precision, except on *Coke* and *Sylvester*. However, for *Coke*, the precision of MCT is the highest in the range of [0, 80]. For *Boy*, *Jogging-1*, *Girl*, *Football* and *Basketball*, MCT is stable and still the first algorithm to reach 100% precision. In the range of approximately [0, 15], the precision of MCT is lower than those of DFT and SCM on *Basketball* and lower than those of DFT, CT, MTT, ORIA, and L1APG on *Football*, while other algorithms suffer from drifting and struggle to reach 100% precision. In particular, for *Football*, when the threshold is larger than 18, MCT first reaches 100% precision at a high rate and later increases steadily. For *Skating1*, in the range of approximately [0, 20], MCT has similar performance to ASLA and SCM, but the precision of MCT is higher than those of the other algorithms. However, when the threshold is larger than 20, the precision of MCT is the highest. Table 4 lists the average precisions of the eight comparison algorithms and our algorithm MCT for a threshold of 20. The average precision of MCT is higher than those of the other eight algorithms on most of the test sequences. Thus, MCT outperforms the other comparison algorithms.

3. Success rate comparison

**Figure 8:**
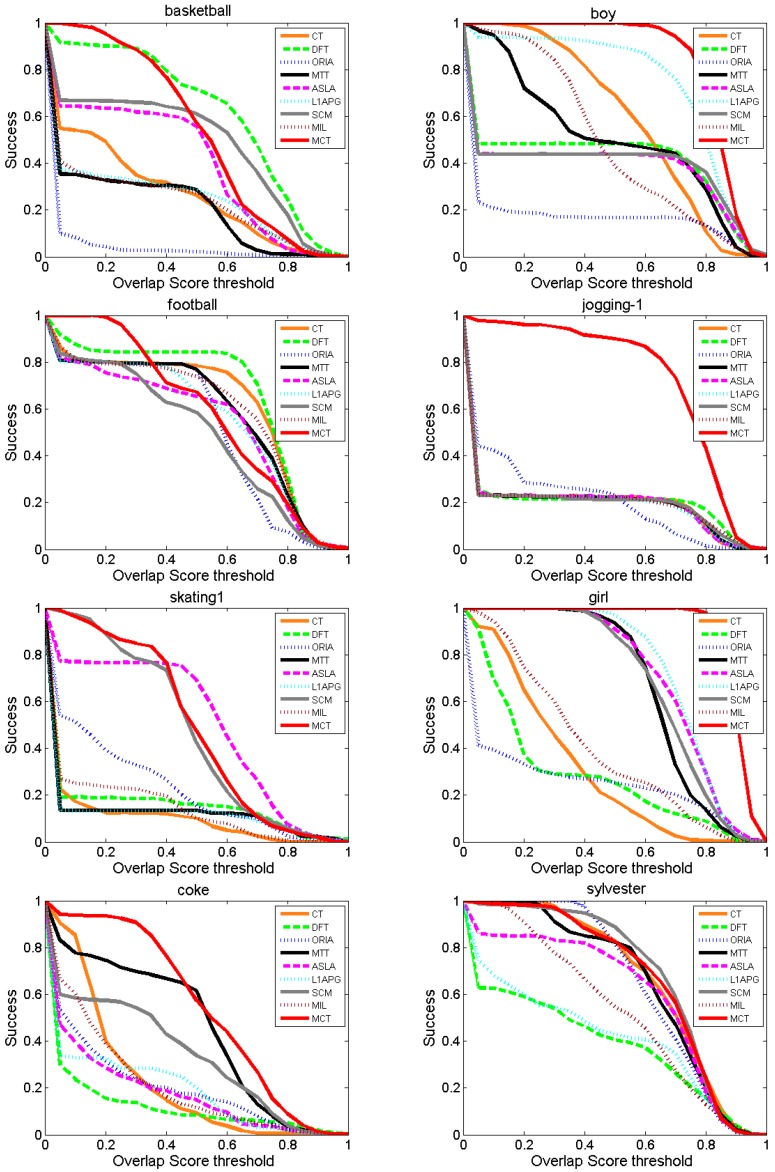
Success rate comparison of the algorithms.

Figure 8 shows the success rates of each algorithm at different values of the threshold λ within [0, 1]. The area under the curve (AUC) of Figure 8 is utilized to evaluate the algorithms. For *Boy*, *Jogging-1*, *Skating1*, *Coke* and *Girl*, the AUC of MCT is the highest compared with the other algorithms. Especially for *Boy*, *Jogging-1* and *Girl*, the success rate of MCT decreases stably at a low rate. For *Basketball*, the performance of MCT is similar to that of the DFT algorithm, but the performance of MCT is better than that of DFT at thresholds lower than approximately 0.3. For *Sylvester*, the success rate of MCT is the highest at thresholds in [0, 0.3] and is close to 1, and the performance of MCT is similar to those of CT, SCM and ORIA.

Table 5 lists the average success rate (AUC) of eight comparison algorithms and MCT. The MCT algorithm achieves the highest average success rate on *Boy*, *Jogging-1*, *Skating1*, *Coke* and *Girl*. According to the comparison results, MCT outperforms the other algorithms on the metric of success rate.

### 4.2. Qualitative Evaluation

**Figure 9:**
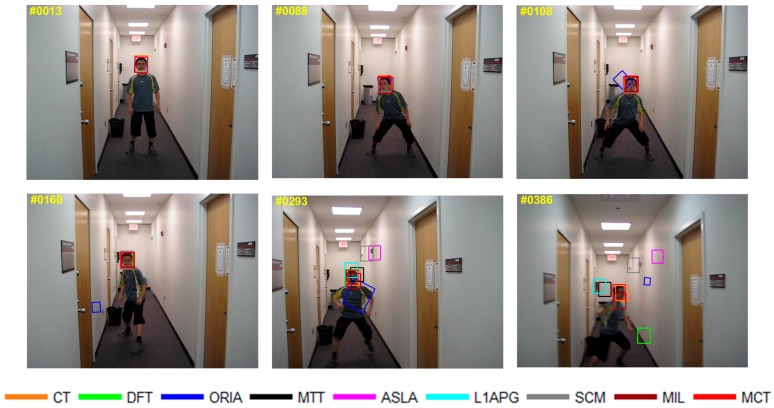
Tracking results on *Boy*.

**Figure 10:**
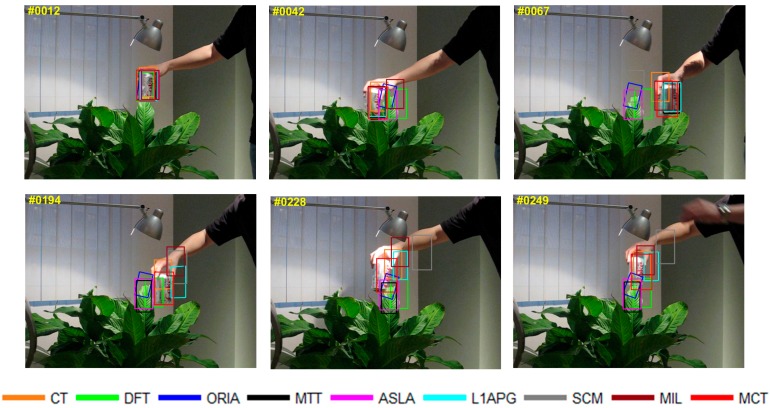
Tracking results on *Coke*.

**Figure 11:**
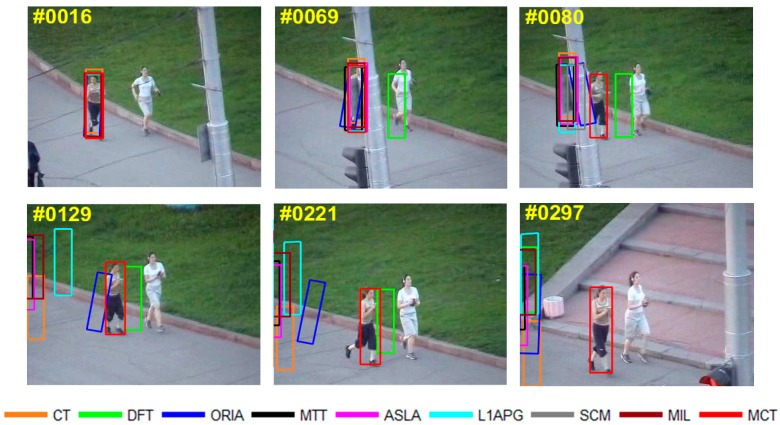
Tracking results on *Jogging-1*.

**Figure 12:**
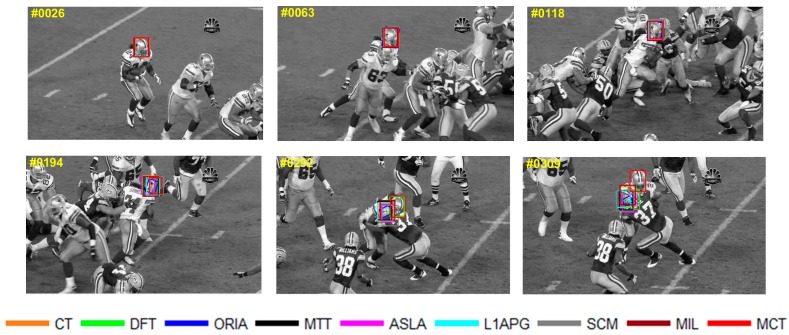
Tracking results on *Football*.

**Figure 13:**
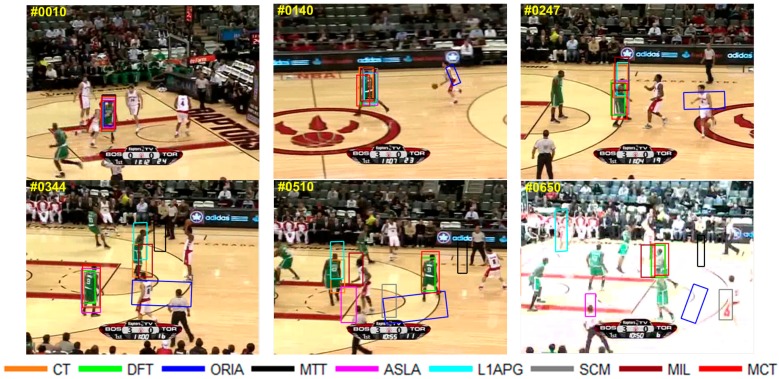
Tracking results on *Basketball*.

**Figure 14:**
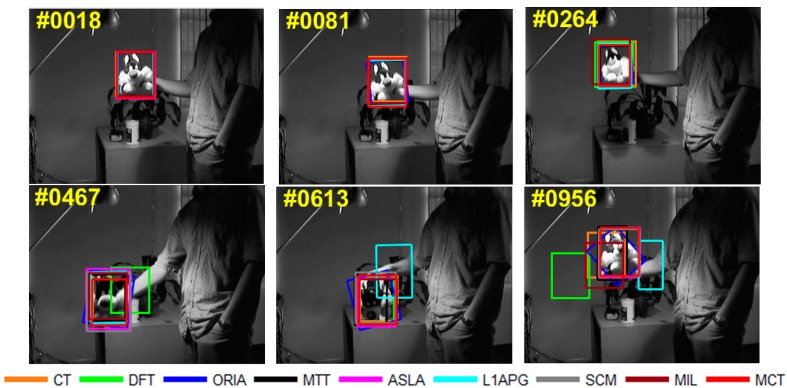
Tracking results on *Sylvester*.

**Figure 15:**
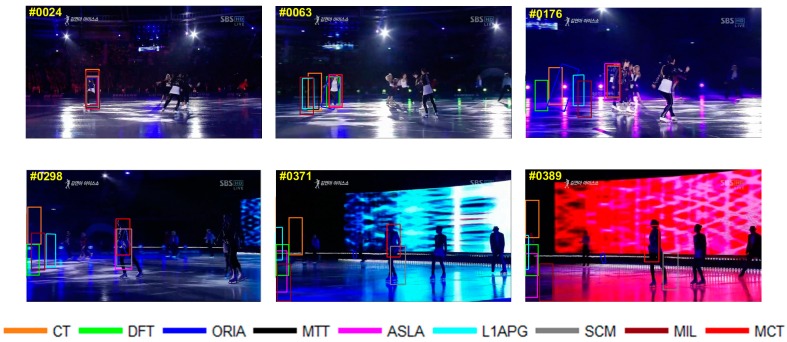
Tracking results on *Skating1*.

**Figure 16:**
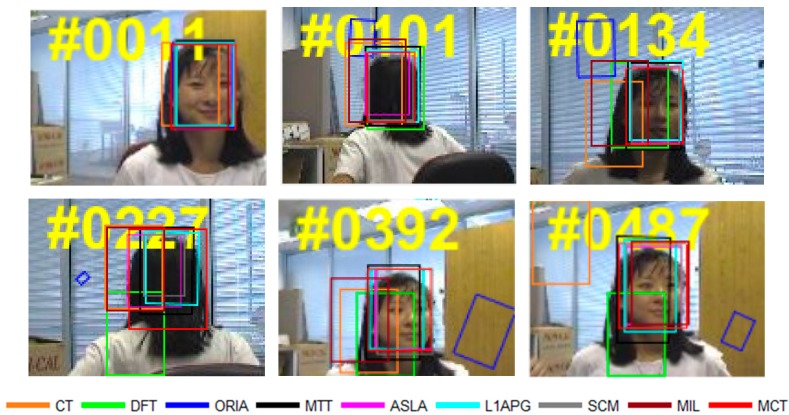
Tracking results on *Girl*.

The qualitative analysis results of the algorithms on eight test videos are shown in Figure 9, Figure 10, Figure 11, Figure 12, Figure 13, Figure 14, Figure 15 and Figure 16. All the problems we concerned are covered in these video sequences, fast motion and scale variation, partial or full occlusion, the deformation and illumination variation. We analyze the performance of algorithms on eight specific video sequences with different problems one by one.

1. Fast motion

As shown in Figure 9, the content of the video sequence *Boy* is a dancing boy. In the process of dancing, the target shakes randomly with fast motion as shown from 88th to 386th. The boy’s appearance blurs during the fast motion. The tracking box of ORIA cannot keep up with the fast motion of the target and the tracking drift occurs in 108th. In the 160th frame, the target moves backwards, the tracking box of ORIA loses the target completely in 160th. In the 293rd frame, the boy is squatting down, the tracking boxes of SCM and ALSA cannot keep up with the variation of the target, which leads to tracking failure after 293rd. Besides, the tracking boxes of MTT, L1APG and MIL have tracking drift in 293rd. As tracking continues, CT, MTT, L1APG, SCM, MIL and other algorithms also cannot keep up with the target motion. Only MCT is able to properly track the target.

For *Coke*, a can held by a man moves fast within a small area in the complex background with the dense bushes. Figure 10 shows the tracking results on Coke. In the 42nd frame, because of occlusion by plants, the algorithms DFT, L1APG MIL and ASLA have tracking drifts to different extents, while MCT and MTT perform well. Then the target keeps moving fast from the 42nd to the 67th frame. The motion directions vary rapidly, which results in tracking drifts with the L1APG, MTT and CT algorithms in 67th. The tracking box of DFT, ORIA and ASLA lose the target completely in 67th, while the MCT and MIL algorithms can accurately track the target in the presence of fast motion. After 194th frame, all algorithms except MCT fail to track the target. Throughout the entire process of fast motion, the MCT algorithm can stably track the target.

Our algorithm, MCT, performs notably well on videos that involve fast motion. This is because the MCT algorithm takes into account motion consistency so that targeted candidate samples can be calculated after predicting the target’s possible motion state during fast motion, which can possibly cover the region where the target may appear. In addition, the position factor is proposed to decrease the influence of secondary samples, and an adaptive strategy is utilized to address the change in the target’s appearance brought about by fast motion. All of these factors guarantee highly stable and accurate tracking.

2. Partial and full occlusion

Two ladies are jogging in the video *Jogging-1*. As shown in Figure 11, partial and full occlusions are caused by the pole during the jogging from the 69th to 80th frame. In the 69th frame, the DFT algorithm fails to track the target first when partial occlusion occurs by the pole and the DFT algorithm turns to track the jogger with white shirt. In the 80th frame, after the jogger we tracked appears without partial or full occlusion, all the algorithms lose the target completely except MCT. In the following frames, while the other algorithms fail to track the target, MCT can stably track the target until the occlusion completely disappears in the 129th frame. The tracking box of DFT has tracking drift in the 221st frame. In the 297th frame, all the algorithms except MCT have no overlap with the true target.

The tracking results on *Football* are shown in Figure 12. For *Football*, occlusion occurs when football players compete in the game. The players are crowded in the presence of the ball robbing. And the partial or full occlusion will occur in the situation of ball robbing. In the 118th frame, the players are crowded in the same place and the target we tracked is in the middle of the crowds, then the partial occlusion occurs at the head of the target, which causes confusion of different football players. As the competition goes, the algorithms have a tracking drift in the frame 194th. In 292nd, only MCT and DFT can accurately track the target under the full occlusion. The tracking box of L1APG, ASLA, MIL and other algorithms lose the target and turn to track another player close to the target we tracked. When the occlusion disappears in the 309th frame, only MCT can still track the target.

In MCT, the occlusion factor is proposed in the local representation. When occlusion occurs, the background information of the target increases, and the occlusion factor will change correspondingly. Partial and full occlusions can be rectified based on the occlusion factor in appearance model.

3. Deformation and illumination variation

For *Basketball*, the target *No.9* basketball player’s appearance varies in the game because of inconstant motion. Moreover, illumination changes because reporters take photos, especially when players are shooting. The tracking results of the nine algorithms are shown in Figure 13. As we can see, when the player starts to run, almost all the tracking boxes have tracking drift in the frame 140th, and ORIA lose the target completely. When the deformation occurs in 247th, ORIA, MTT, L1APG, CT and MIL fail to track the target. Only DFT and MCT can track the target with a slight tracking drift. In the 344th frame, the player turns around, while MCT, SCM, DFT, and ASLA succeed and other algorithms lose the true target. In the 510th frame, when the player moves to spectator, all the algorithms fail to track except MCT and DFT. However, DFT has tracking drifts. When illumination changes in the 650th frame, only MCT and DFT can track the target.

For *Sylvester*, a man shakes a toy under the light. Within the whole process, as shown in Figure 14, illumination and deformation of the toy change, and the plane rotates inside and outside occur. The tracking boxes have drift when the toy begins to move under the light in the frame 264th. In the 467th frame, the toy is lower its head, in such a situation, the algorithm DFT almost lose the target. Then in the 613rd frame, when the toy has rotation, the algorithm L1APG fails to track the target and other algorithms have tracking drift. In the 956th frame, the toy is rotating under the light, and more algorithms such as DFT and L1APG lose the target. However, MCT performs well under the circumstances with deformation and illumination especially shows robustness within illumination decreasing between the 264th to the 467th frame.

First, MCT is based on particle filtering framework, so that to some extent diversity of candidate samples can resolve elastic deformation. Secondly, holistic representation and local representation are utilized in the appearance model, which shows great robustness by enabling holistic and local representation to capture the changes of target’s deformation and illumination variation. Finally, when models are being updated, appropriately updating positive and negative templates can also adapt to the changes of appearance and environment.

4. Scale variation and rotation

For *Skating1*, a female athlete is skating on ice, and the athlete continuously slides so the relative distance to camera changes continuously, thus the athlete has a scale variation in the scene. Besides, the athlete is dancing in the process, thus the athlete also has a rotation in the scene. It can be seen in Figure 15, from the 24th frame to the 176th frame, the size of the target becomes smaller, and the algorithms SCM, ASLA and MCT can track the target. And CT, DFT, ORIA, L1APG and MIL lose the true target in the frame 176th. From the 176th to the 371st frame, the target moves to the front of the stage and becomes larger with rotation on the stage, and almost all the algorithms fail to track except MCT and SCM. But SCM has tracking drift. In the 389th frame, SCM loses the athlete, and only MCT can track the target.

For *Girl*, a woman sitting on the chair varies her position and facing direction by rotating the chair. It can be seen in Figure 16, the size of the target changes from the 11st to the 392nd frame with rotation on the chair. Because of the chair’s rotation, the size of the target becomes smaller in the 101st frame compared with that in the 11st frame. After that, the girl turns around and becomes larger in 134th. In this process, all the algorithms have tracking drift, and ORIA loses the target completely from 101st to 487th. When the target turns around from 101st to 134th, the tracking boxes of ORI, CT and MIL lose the target. Although the appearance of the target changes promiscuously from 227th to 487th, MCT performs well under this circumstance and shows robustness and accuracy compared with other algorithms.

Based on particle filter, six affine parameters including parameters indicate scaling variation and rotation are used in MCT to represent the candidate samples. Thus, the diversity of scaling can be ensured in prediction of candidate samples, which could adapt to the scene of scale variation.

### 4.3. Discussion

In this part, we further illustrate the selection of related means in MCT and comparison of the tracking results and computational complexity between MCT and SCM.

#### 4.3.1. Discussion about the Number of Particles

**Figure 17:**
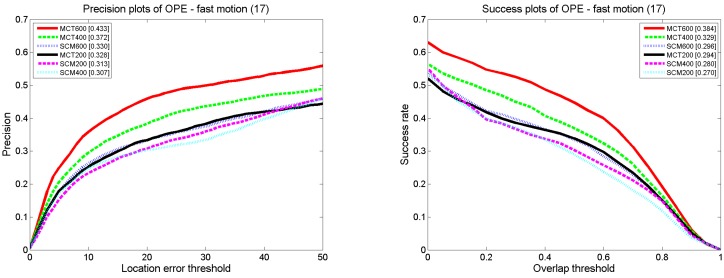
Comparison between MCT and MCT within different number of particles.

Most of the tracking methods within the particle filter framework exploit the Gaussian distribution model to predict candidate samples. Our algorithm, MCT, predicts targeted candidate samples by estimating the target motion direction and distance; in addition, the position factor is defined to assign weights to candidate samples in different positions. To prove the superiority of the MCT algorithm in terms of resource saving, we show that the smaller number of samples predicted by our method can achieve better performance with fewer particles compared with other algorithms. To do so, we conduct an experiment on MCT and SCM with 200, 400, and 600 particles. The test sequence consists of 17 videos from the CVPR2013 benchmark datasets with fast motion properties. The results are shown in Figure 17.

The success rate and tracking precision of MCT with 600 and 400 particles are higher than those of SCM with 600 particles; the success rate and tracking precision of MCT with 200 particles are higher than those of SCM with 400 and 200 particles. MCT can achieve better performance with fewer particles compared with SCM. In this paper, we predict the target state and incorporate the target state information into the algorithm. More candidate samples are obtained in the position where the target may appear with a larger probability, and weights are assigned to all of the candidates according to the position factor. Thus, the impact of invalid samples on the computation decreases and better performance can be achieved with fewer particles.

#### 4.3.2. Discussion of Position Factor

**Figure 18:**
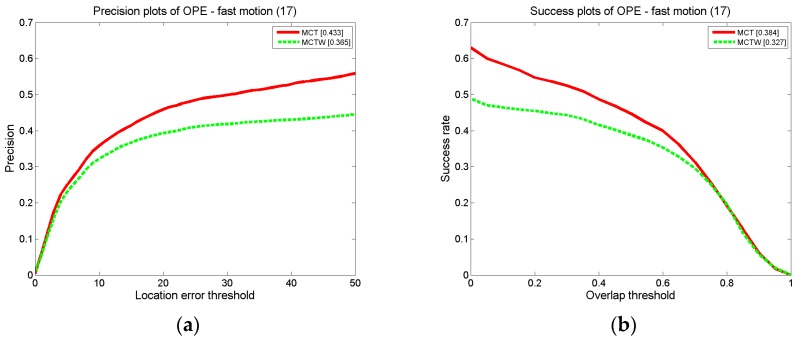
The precision plot (**a**) and the success rate plot (**b**) on MCT and MCTW for FM.

According to the motion consistency, the states of the target in successive frames are correlated. Therefore, the probability of the target appearing in each position is different, and the importances of candidate samples located in different positions also differ. Thus, a double Gaussian probability model is proposed due to the motion consistency in successive frames to simulate the target state, based on which the position factor is proposed to assign weights to candidate samples to represent the differences between candidate samples with different locations. To demonstrate the significant impact of the position factor, an experiment is conducted with 600 particles on MCT and MCT without the position factor (MCTW), and the test sequence consists of 17 videos from the CVPR2013 benchmark datasets with fast motion properties. The results are shown in Figure 18. According to the experimental results, the precision of MCT is 6.8% higher than that of MCTW, and the success rate is 6.7% higher than that of MCTW. Thus, the position factor improves the tracking precision and success rate.

#### 4.3.3. Discussion of Computational Complexity

The computational complexity is an important evaluation factor. We also evaluate the computational complexity of the MCT algorithm. Since both MCT and SCM algorithms are based on the particle filter, we will provide the complexity analysis between these two algorithms. The computational complexity will be evaluated by the tracking speed. The higher the tracking speed, the lower the computational complexity of the algorithm. The experiments of computational complexity are all implemented on the computer in our lab, the computer with 3.20 GHz CPU, 4.0 GB RAM and Windows 10 operating system. And the results on eight specific videos are shown in Table 6. In addition, the tracking speed of MCT is close to that of SCM. Simultaneously, the tracking precision of MCT can achieve 10% increment on fast motion compared with SCM as shown in the results of quantitative evaluation. This is because the target state prediction, the position factor and the target velocity prediction were developed in state transition model, appearance model and updating scheme respectively compared with SCM.

In addition, the experimental results show that the computational complexity of the tracker is related to the target size. It is because in appearance model, the target is divided into patches, which will be represented sparely by the dictionary. In fact, the tracking speed is also influenced by other factors, such as the different update frequencies in videos with different scenes. Accordingly, the computational complexity of the tracker will decrease if the size of target becomes smaller, meanwhile the fluctuation will still exist.

## 5. Conclusions

In this paper, we propose an object tracking algorithm based on motion consistency. The target state is predicted based on the motion direction and distance and is later merged into the state transition model to predict additional candidate samples. The appearance model first exploits the position factor to assign weights to candidate samples to characterize the different importance of candidate samples in different positions. At the same time, the occlusion factor is utilized to rectify the similarity in the local representation and obtain the local response, and the candidates are represented sparsely by positive and negative templates in the holistic representation to obtain the holistic response. The tracking result is determined by the position factor with the local and holistic responses of each candidate. The adaptive template updating scheme based on target velocity prediction is proposed to adapt to appearance changes when the object moves quickly by updating the dynamic positive templates and negative templates continuously. According to qualitative and quantitative experimental results, the proposed MCT has good performance against the state-of-the-art tracking algorithms, especially in dealing with fast motion and occlusion, on several challenging video sequences.

## Figures and Tables

**Table 1:** Basic information of the eight state-of-the-art tracking algorithms.

Algorithm	State Transition Model	Appearance Model
L1APG	Constant velocity model	Holistic representation
MTT	Gaussian distribution model	Holistic representation
ASLA	Gaussian distribution model	Local representation
SCM	Gaussian distribution model	Holistic & Local representation
MIL	Dense sampling search	Holistic representation
CT	Dense sampling search	Holistic representation
DFT	Local optimum search	Holistic representation
ORIA	Local optimum search	Holistic representation

**Table 2:** Basic information of the eight test videos.

Video	Image Size	Target Size	Main Confronted Problems
*Coke*	640 × 480	48 × 80	Fast motion
*Boy*	640 × 480	35 × 42	Fast motion
*Football*	624 × 352	39 × 50	Occlusion
*Jogging-1*	352 × 288	25 × 101	Occlusion
*Girl*	128 × 96	31 × 45	Scale variation and rotation
*Skating1*	640 × 360	34 × 84	Scale variation and rotation
*Basketball*	576 × 432	34 × 81	Deformation and illumination variation
*Sylvester*	320 × 240	51 × 61	Illumination variation

**Table 3:** Average center location error of the algorithms.

Video	CT	DFT	ORIA	MTT	ASLA	L1APG	SCM	MIL	MCT
*Basketball*	89.11	18.03	152.09	106.80	82.63	137.53	52.90	91.92	12.79
*Boy*	9.03	106.31	132.90	12.77	106.07	7.03	51.02	12.83	2.10
*Football*	11.91	9.29	13.61	13.67	15.00	15.11	16.30	12.09	8.86
*Jogging-1*	92.49	31.44	50.43	108.03	104.58	89.52	132.83	96.34	5.38
*Skating1*	150.44	174.24	69.62	293.34	59.86	158.70	16.38	139.38	15.38
*Girl*	18.85	23.98	27.65	4.29	3.28	2.80	2.60	13.67	1.42
*Coke*	40.49	70.70	49.96	29.98	60.17	50.45	56.81	46.72	19.44
*Sylvester*	8.556	44.883	5.683	7.554	15.227	26.244	7.968	15.504	8.030
Average	52.610	59.859	62.743	72.054	55.852	60.923	42.101	53.557	9.175

Red is the best, blue is the second, green is the third.

**Table 4:** Average precision of the algorithms.

Video	CT	DFT	ORIA	MTT	ASLA	L1APG	SCM	MIL	MCT
*Basketball*	0.453	0.847	0.087	0.336	0.582	0.329	0.640	0.398	0.869
*Boy*	0.906	0.477	0.285	0.868	0.440	0.926	0.512	0.868	0.974
*Football*	0.877	0.903	0.860	0.860	0.846	0.845	0.834	0.875	0.907
*Jogging-1*	0.244	0.694	0.516	0.249	0.253	0.266	0.256	0.253	0.942
*Skating1*	0.231	0.189	0.529	0.160	0.717	0.217	0.833	0.271	0.843
*Girl*	0.808	0.757	0.722	0.953	0.963	0.968	0.969	0.960	0.981
*Coke*	0.594	0.313	0.503	0.703	0.404	0.510	0.528	0.532	0.819
*Sylvester*	0.910	0.610	0.939	0.920	0.844	0.735	0.916	0.844	0.915
Average	0.628	0.599	0.555	0.631	0.631	0.560	0.686	0.625	0.906

Red is the best, blue is the second, green is the third.

**Table 5:** Average success rate of the algorithms.

Video	CT	DFT	ORIA	MTT	ASLA	L1APG	SCM	MIL	MCT
*Basketball*	0.278	0.604	0.072	0.223	0.398	0.256	0.475	0.247	0.526
*Boy*	0.587	0.418	0.189	0.502	0.389	0.725	0.397	0.491	0.818
*Football*	0.607	0.651	0.513	0.575	0.533	0.553	0.489	0.585	0.580
*Jogging-1*	0.212	0.218	0.221	0.211	0.213	0.208	0.212	0.214	0.717
*Skating1*	0.123	0.172	0.242	0.144	0.501	0.143	0.471	0.161	0.487
*Girl*	0.318	0.293	0.263	0.657	0.701	0.719	0.673	0.403	0.880
*Coke*	0.241	0.142	0.215	0.449	0.192	0.203	0.342	0.224	0.539
*Sylvester*	0.658	0.391	0.642	0.641	0.590	0.413	0.677	0.528	0.659
Average	0.378	0.361	0.295	0.425	0.440	0.403	0.467	0.357	0.650

Red is the best, blue is the second, green is the third.

**Table 6:** Tracking speed comparison of the algorithms.

Tracking Speed (FPS)	*Coke*	*Sylvester*	*Skating1*	*Basketball*	*Jogging-1*	*Football*	*Boy*	*Girl*	Average
SCM	0.3138	0.3390	0.2780	0.3801	0.3759	0.4292	0.3512	0.3171	0.3480
MCT	0.3089	0.2951	0.2557	0.3146	0.3345	0.3417	0.3498	0.3683	0.3211

(The target size decreases from *Coke* to *Girl.*)

## Supplementary Materials

The following are available online at http://www.mdpi.com/1424-8220/18/2/572/s1, Video S1: Figure 9-Boy, Video S2: Figure 10-Coke, Video S3: Figure 11-Jogging-1, Video S4: Figure 12-Football, Video S5: Figure 13-Basketball, Video S6: Figure 14-Sylester, Video S7: Figure 15-Skating1, Video S8: Figure 16-Girl.
